# Supplementary material for: Effect of virtual reality (VR) technology on anxiety control and acrophobia reduction: A randomized controlled trial in Iran
Source: Glob Ment Health (Camb). 2026 Feb 27;13:e93. doi: 10.1017/gmh.2026.10160 (PMC13184657; doi:10.1017/gmh.2026.10160)
Supplement: Breen et al. supplementary material [file S2054425126101605sup001.docx]

| Row | **Items** | Completely disagree | Disagree | No idea | Agree | Completely agree |
| --- | --- | --- | --- | --- | --- | --- |
| 1 | When I go to high place, I do not dare to look down. |  |  |  |  |  |
| 2 | I get sick of seeing heights even in TV movies. |  |  |  |  |  |
| 3 | When I climb a high place, I tremble involuntarily. |  |  |  |  |  |
| 4 | I can not help someone who is stuck in a high place. |  |  |  |  |  |
| 5 | When I am in a high place, my thoughts are disturbed and I can not concentrate. |  |  |  |  |  |
| 6 | I do not like high jump scenes in movies, because it is usually confusing. |  |  |  |  |  |
| 7 | When I go up the ladder, I feel confused. |  |  |  |  |  |
| 8 | When I have to go high, my arms and legs become weak. |  |  |  |  |  |
| 9 | Most nights I have nightmares of falling from heights. |  |  |  |  |  |
| 10 | Seeing the height makes me nervous and I lose control of my behavior. |  |  |  |  |  |
| 11 | I do not like exercises that are done at height (parachuting, paragliding, etc.). |  |  |  |  |  |
| 12 | I'm afraid of traveling by plane. |  |  |  |  |  |
| 13 | Even in my mind I am scared when I think of heights. |  |  |  |  |  |
| 14 | When I am in a high place, my appetite goes blind. |  |  |  |  |  |

Apendix 1. Acrophobia Questionnaire (Fear of high places)
